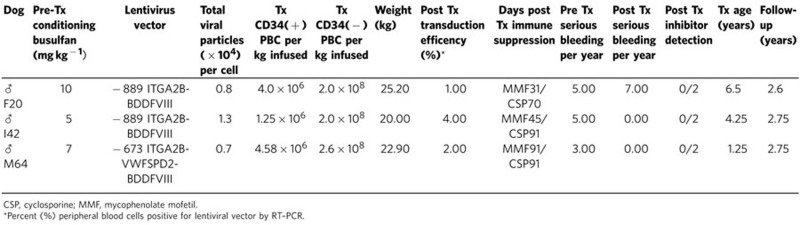# Platelet-targeted gene therapy with human factor VIII establishes haemostasis in dogs with haemophilia A

**DOI:** 10.1038/ncomms3773

**Published:** 2013-11-19

**Authors:** Lily M. Du, Paquita Nurden, Alan T. Nurden, Timothy C. Nichols, Dwight A. Bellinger, Eric S. Jensen, Sandra L. Haberichter, Elizabeth Merricks, Robin A. Raymer, Juan Fang, Sevasti B. Koukouritaki, Paula M. Jacobi, Troy B. Hawkins, Kenneth Cornetta, Qizhen Shi, David A. Wilcox

**Affiliations:** 1 Department of Pediatrics, Medical College of Wisconsin, Milwaukee, Wisconsin 53226, USA; 2 Children’s Research Institute, Children’s Hospital of Wisconsin, Milwaukee, Wisconsin 53226, USA; 3 MACC Fund Research Center, Milwaukee, Wisconsin 53226, USA; 4 Plateforme Technologique et d’Innovation Biomédicale, Hôpital Xavier Arnozan, Pessac 33604, France; 5 Institut de Rythmologie et de Modélisation Cardiaque, Hôpital Xavier Arnozan, Pessac 33600, France; 6 Department of Pathology and Laboratory Medicine, University of North Carolina, Chapel Hill, North Carolina 27516, USA; 7 Biomedical Resource Center, Medical College of Wisconsin, Milwaukee, Wisconsin 53226, USA; 8 Blood Research Institute, BloodCenter of Wisconsin, Milwaukee, Wisconsin 53226, USA; 9 Department of Medical and Molecular Genetics, Indiana University School of Medicine, Indianapolis, Indiana 46202, USA

## Abstract

It is essential to improve therapies for controlling excessive bleeding in patients with haemorrhagic disorders. As activated blood platelets mediate the primary response to vascular injury, we hypothesize that storage of coagulation Factor VIII within platelets may provide a locally inducible treatment to maintain haemostasis for haemophilia A. Here we show that haematopoietic stem cell gene therapy can prevent the occurrence of severe bleeding episodes in dogs with haemophilia A for at least 2.5 years after transplantation. We employ a clinically relevant strategy based on a lentiviral vector encoding the *ITGA2B* gene promoter, which drives platelet-specific expression of human FVIII permitting storage and release of FVIII from activated platelets. One animal receives a hybrid molecule of FVIII fused to the von Willebrand Factor propeptide-D2 domain that traffics FVIII more effectively into α-granules. The absence of inhibitory antibodies to platelet-derived FVIII indicates that this approach may have benefit in patients who reject FVIII replacement therapies. Thus, platelet FVIII may provide effective long-term control of bleeding in patients with haemophilia A.

## Abstract (web summary)

Haemophilia is a genetic bleeding disorder associated with a deficiency in the coagulation factor VIII. Here, the authors use gene therapy to achieve stable overexpression of factor VIII in platelets of dogs with haemophilia A, preventing the occurrence of severe bleeding episodes for over 2.5 years.

## Graphical abstract

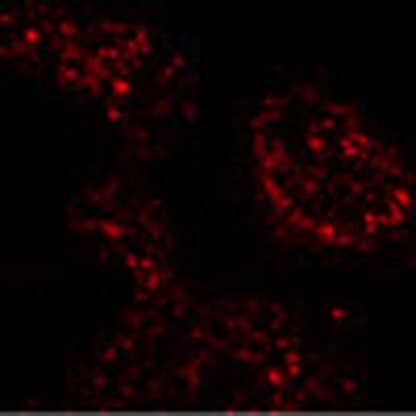

There are several well characterized inherited genetic defects that affect various aspects of platelet function and blood coagulation that usually manifest themselves clinically as a failure to control bleeding^1^. Of these, haemophilia A is a common haemorrhagic disorder (1:10,000 males) linked to quantitative and/or qualitative defects in the plasma protein coagulation Factor VIII (FVIII)^2^,^3^. A canine model for haemophilia A exists, which results from a genetic mutation causing a large inversion of the *FVIII* gene (that resembles a molecular genetic defect found in ≈40% of humans with the severe haemophilia A)^4^. Likewise, canine haemophilia A is essentially identical to the human disease in its clinical presentation characterized by severe-intermittent episodes of joint bleeding and haemorrhage. Protein replacement therapy is the most common treatment of severe bleeding episodes for haemophilia A but it has been confounded by the formation of inhibitory antibodies to transfused human FVIII in 30% of patients^5^,^6^. Similarly, 100% of dogs utilized from the Chapel Hill colony for this study develop inhibitory antibodies after being transfused with human FVIII (ref. 7), albeit severe bleeding is successfully treated with canine FVIII supplements. Thus, canine haemophilia A appears to be an ideal system to determine whether platelets can be used successfully to deliver human FVIII to the site of a vascular injury as a feasible approach to improve haemostasis within a ‘large-animal’ model of haemophilia A with the ability to form inhibitory antibodies to human FVIII.

Recent reports indicate that improved therapies are evolving to control excessive bleeding in patients with severe haemorrhagic disorders including the use of new therapeutic agents and novel gene transfer vectors that target production of deficient coagulation proteins within the liver^8^,^9^,^10^,^11^. However, we and others hypothesized that an autologous transplant of hematopoietic stem cells transduced with a gene encoding FVIII may be an ideal approach for correction of haemophilia A within humans^12^,^13^. As activated blood platelets mediate the primary response to vascular injury by adhering to a wound site and secreting biologically active proteins^14^, we hypothesized that synthesis and storage of FVIII within platelets may be an ideal strategy for providing a continuous, locally inducible treatment for maintaining haemostasis for haemophilia A. The use of platelet FVIII to maintain haemostasis was shown to be a successful approach for correcting murine haemophilia A^15^,^16^, although a suitable protocol employing platelet FVIII for human gene therapy remains to be shown as feasible within a large animal model for haemophilia A. Thus, the current investigation expands significantly upon platelet gene transfer technology by demonstrating the development of a clinically relevant strategy for transferring genes into G-CSF cytokine-mobilized peripheral blood stem cells (G-PBC) to improve haemostasis for canine haemophilia A.

To accomplish this goal, a fragment of the *integrin αIIb* (*ITGA2B)* gene promoter known to drive megakaryocyte-specific gene transcription^17^ was employed for ectopic expression of human B-domain-deleted Factor VIII (BDDFVIII) because megakaryocyte-targeted expression of *ITGA2B* and *integrin β3* (*ITGB3*) genes was previously shown to be useful for hematopoietic stem cell gene therapy leading to correction of platelet function in dogs and mice affected with the inherited bleeding disorder Glanzmann thrombasthenia (GT)^18^,^19^. Second, herein we show that the VWFSPD2 can help traffic BDDFVIII efficiently into platelet α-granules (which are secreted from activated platelets at the vascular injury site). Finally, transient post-transplant immune suppression is employed in this study to induce tolerance to platelet BDDFVIII because this approach was previously shown to be useful in other gene transfer studies^7^,^10^,^19^.

## Results

### Platelet-targeted lentiviral vector design and strategy

**Figure 1:**
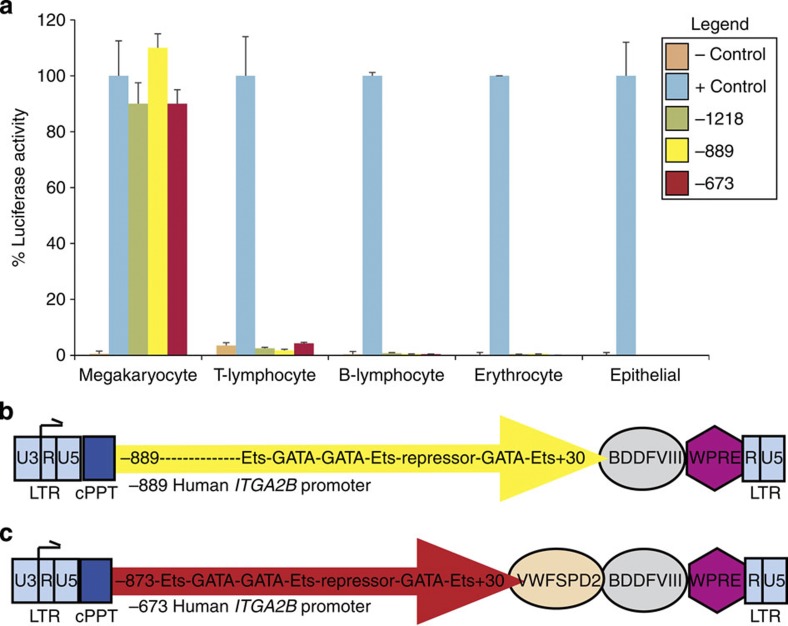
Platelet-targeted lentiviral vector design. (**a**) *ITGA2B* gene promoter fragments direct megakaryocyte-specific expression in luciferase reporter assay. Human pro-megakaryocytic, lymphocytic, erythrocytic and epithelial cell lines are transfected with a luciferase reporter construct under the control of one of three fragments of the human *ITGA2B* gene promoter beginning at either nucleotide −1218(green), −889(yellow) or −673(red) in-frame with the luciferase gene. A luciferase construct without a gene promoter served as a negative (−)control (peach), whereas a construct with a tissue-nonspecific gene promoter of the cytomegalovirus (CMV) is a positive (+)control (blue) arbitrarily assigned a value of 100% luciferase activity. Lysates of each cell line (*x* axis) were measured in duplicate on a luminometer to report the percent (%) luciferase activity (*y* axis) for each construct within each cell line. Cells were co-transfected with plasmid constructs encoding the marker gene, β-galactosidase, under the under the control of the CMV promoter to correct for variations in transfection efficiency. Shown is one representative of nine experiments where the error bars represent the s.d. from the mean value of measurements made in duplicate. (**b**) −889*ITGA2B*-BDDFVIII-WPTS lentiviral vector diagram. Shown is the region between the 5′-long terminal repeat (LTR) (left, blue box) and 3′-LTR without a U3 enhancer/promoter (right, blue box) to permit the *ITGA2B* promoter (yellow arrow) to direct megakaryocyte-specific synthesis of BDDFVIII (grey circle). The −889*ITGA2B* promoter binds GATA and Ets transcription factors for high-level gene expression in megakaryocytes and a repressor region inhibits gene expression in other lineages (yellow arrow). The central polypurine tract (cPPT, navy) and woodchuck hepatitis virus postregulatory element (WPRE, purple diamond) enhance efficiency of transgene expression. (**c**) −673*ITGA2B*-VWFSPD2-BDDFVIII-WPTS lentiviral vector diagram. The backbone of this vector is the same as described in **b**, except a smaller −673*ITGA2B* promoter (red arrow) was fused to DNA encoding the human VWF signal peptide (SP) and D2 domain (peach circle) to traffic BDDFVIII (grey circle) directly to platelet α–granules.

A luciferase reporter assay revealed that fragments of the full-length human *ITGA2B* gene promoter permitted comparable platelet-specific gene transcription (Fig. 1a). Three different *ITGA2B* promoter fragments (−1218, −889 and −673) directed similar levels of luciferase activity within a pro-megakaryocytic cell line. In contrast, *ITGA2B* promoter-driven luciferase activity remained undetectable in the other blood cell lineages and an epithelial cell line. Each *ITGA2B* promoter encodes Ets and GATA factors permitting a high level of megakaryocyte gene transcription and a repressor region that inhibits expression within other lineages^20^. As a result, two lentiviral gene transfer vectors were tested for optimal hematopoietic stem cell transduction efficiency and the ability to improve haemostatic function with platelet-derived BDDFVIII in haemophilia A dogs to develop a novel strategy that may prove beneficial for human gene therapy. Two dogs received an infusion of G-PBC transduced with a lentiviral vector encoding a fragment beginning at −889 nucleotide of the human *ITGA2B* promoter shown to be capable of directing megakaryocyte-specific transcription of BDDFVIII (Fig. 1b)^21^. Although FVIII is absent from platelets under normal conditions, this approach proved successful for storing viable BDDFVIII in platelet progeny derived from tissue-cultured human CD34+G-PBC^12^ and lentiviral vector-transduced bone marrow transplanted into haemophilia A mice^16^. One dog received an infusion of G-PBC transduced with a novel lentiviral vector encoding the shortest fragment of the *ITGA2B* promoter (−673) designed to induce megakaryocyte-specific expresson of a hybrid molecule of BDDFVIII fused to the von Willebrand factor (VWF) propeptide signal peptide and D2 domain (SPD2) to facilitate trafficking of BDDFVIII into the α-granule compartment (Fig. 1c)^22^,^23^. VWF is a normal α-granule constituent in human platelets (albeit absent in canine platelets)^24^ that serves as a carrier protein of FVIII in human and canine plasma^25^.

### Strategy for hematopoietic stem cell gene therapy

To design a clinically relevant protocol, canine hematopoietic stem cells were mobilized from the bone marrow into the peripheral blood with canine cytokines (cG-CSF and cSCF) and G-PBC apheresis was performed without adverse incidents identical to previous studies using GT dogs^19^,^26^. Mononuclear lymphocytes were isolated with Ficoll-Paque Plus from the apheresis product and then canine CD34 antigen-positive (CD34+) cells were purified by immunomagnetic selection^27^. Table 1 summarizes the conditions for autologous transplant of three haemophilia A dogs transfused with ~3 × 10^6^ FVIII-transduced CD34+G-PBC per kg of body weight where each target cell was transduced with ~1 × 10^4^ total viral particles/CD34+G-PBC without the use of *ex vivo* or *in vivo* selection for transduced cells (columns 4 and 5).

A nonmyeloablative pre-transplant conditioning regimen was employed to create a niche in the bone marrow for the newly transplanted cells to engraft (Table 1, column 2). The intensity of the conditioning regimen is determined by the level at which the dose becomes toxic to the organs. Earlier studies performed with normal canine models have demonstrated that stable allogeneic mixed donor/host hematopoietic chimerism can be safely established by the administration of a sublethal dose of busulfan (a drug preferentially toxic to hematopoietic stem cells) for pre-transplant conditioning. A recent report also demonstrated successful use of busulfan at 10 mg kg^−1^ for hematopoietic stem cell gene transfer to correct canine leukocyte adhesion deficiency^28^, followed by transient immunosuppression with mycophenolate mofetil (MMF) and cyclosporine (CSP) after major histocompatibility complex identical marrow transplantation^29^. However, this level of pre-transplant conditioning regimen proved inappropriate for animals with haemophilia A, because the first dog (F20) transplanted in the current study required daily supplements with canine (c)FVIII in the form of canine plasma products and recombinant cFVIII for 3 months after G-PBC transplant. Epsilon-aminocaproic acid (EACA) was also infused after G-PBC transplant until human BDDFVIII reached a significant level in platelets. EACA is an effective synthetic inhibitor of the plasmin–plasminogen system and controls subarachnoid haemorrhage, genitourinary bleeding from many causes and dental surgery in haemophiliacs^30^. For comparison, the number of serious bleeding episodes that required treatment with cFVIII supplement has been recorded 1 year before and 2.5 years after G-PBC transplant for each dog (Table 1, columns 10, 11)^31^. As a result of the observation of frequent bleeding events with F20, a milder conditioning regimen consisting of a lower dose of busulfan was given to the next two transplant recipients (I42, 5 mg kg^−1^; M64, 7 mg kg^−1^). In conclusion, all three dogs received transient immune suppression^10^,^19^,^32^ and daily supplements of cFVIII and EACA for 3 months after G-PBC transplant as a standard transplant regimen until readily detectable human BDDFVIII levels were observed in platelets and haemocult tests indicated the absence of GI bleeding^10^,^19^,^32^. Ultimately, we observed that I42 and M64 did not require further FVIII supplements as no severe bleeding episodes occurred for 2.5 years after transplant (Table 1, column 11), which will be discussed in greater detail below.

### Biological studies of platelet FVIII

**Figure 2:**
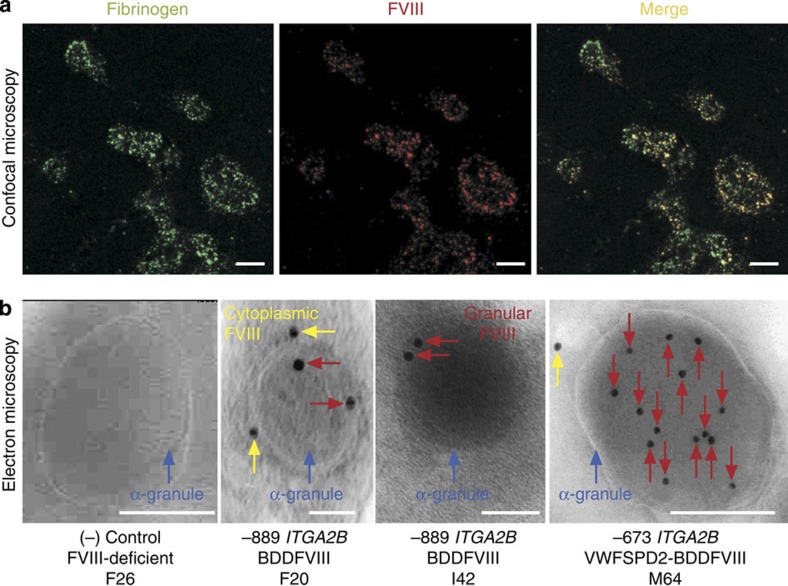
Synthesis and trafficking of BDDFVIII into canine platelet α-granules. (**a**) Confocal microscopy showing co-localization of BDDFVIII and Fg within platelets. Canine CD34+G-PBC were transduced with lentivirions encoding human BDDFVIII followed by transplant haemophilia A dogs. Peripheral blood platelets were isolated from whole blood, fixed, permeabilized and examined by indirect immunofluorescence analysis for Fg and BDDFVIII distribution. Shown is a representative image using a × 10 eye piece and × 100 oil objective and a × 2 digital zoom of platelets isolated from one transplanted animal (I42) from an experiment that was performed seven times on all three dogs and a FVIII-deficient negative control. Fg was visualized with a 1°Ab to this platelet-specific marker for α-granules and a Alexa488-conjugated 2°Ab (left, green). BDDFVIII was detected with 1°Ab to human FVIII and an Alexa568-conjugated 2°Ab (middle, red). BDDFVIII colocalized with Fg is observed when the two images are merged (right, yellow). The white scale bar is 5 μm in length. (**b**) Electron microscopy localized human BDDFVIII directly in α-granules. Shown is a representative image of BDDFVIII absent from an ultrathin cryosection of a single platelet α-granule (blue arrow) from a FVIII-deficient (negative control, F26) when probed with a 1°Ab (301.3) to human BDDFVIII and a 2°Ab-conjugated to 10-nm gold particles. In contrast, BDDFVIII was localized directly within the α-granule (red arrow) and within intracytoplasmic membrane systems (yellow arrow) in representative images from all three experimental dogs (F20, I42 and M64). Interestingly, sections from dog (M64) receiving the −673*ITGA2B*-VWFSPD2-BDDFVIII-transduced G-PBC (VWF-targeting peptide) exhibited a noticably higher concentration of BDDFVIII within mature platelet α-granules. Each sample was cut on a minimum of three separate occasions with a series of ultrathin sections subjected to immunogold labelling and analysis of at least 100 α-granules per sample. The white scale bar is 0.2 μm in length.

Immuno-confocal microscopy was performed to determine if BDDFVIII was being synthesized and stored in platelets following G-PBC transplant. Shown in Fig. 2a are images of the results of microscopic analysis of platelets isolated from one dog (I42) that received an autologous transplant of lentiviral vector-transduced G-PBC, which represents the outcome of analysis of all three dogs (F20, I42 and M64). As expected, there was a punctate staining pattern for a specific marker of platelet α-granules, fibrinogen (Fg) (left panel, green). Interestingly, human BDDFVIII was also detected in a punctate pattern within platelets (middle panel, red). Note that BDDFVIII staining appeared to colocalize frequently with Fg as evident by the appearance of a yellow staining when the left (Fg) and middle panel (BDDFVIII) were overlaid, indicating that both proteins could be stored together within platelet α-granules (right panel, yellow)^12^.

Immunoelectron microscopy was performed to determine whether exogenous BDDFVIII was being transported specifically to platelet α-granules. Immunogold analysis was performed on ultrathin sections of platelets with a 1°Ab to FVIII and a 2°Ab adsorbed on 10-nm gold particles (Fig. 2b). The α-granules appeared normal in size and shape within platelets of FVIII-deficient dogs as well as FVIII transplant recipients (blue arrow). As anticipated, BDDFVIII is absent in platelet α-granules from a FVIII-deficient negative control (left panel). In contrast, BDDFVIII was detected within α-granules (red arrows) and cytoplasm (yellow arrows) of platelets isolated from all three dogs (F20, I42 and M64). This result is consistent with observations reported for ectopic expression of BDDFVIII within platelets of VWF(−/−) transgenic mice affected with von Willibrand disease^33^. Remarkably, −673*ITGA2B*-VWFSPD2-BDDFVIII-transduced platelets from M64 appeared to store the greatest level of BDDFVIII within the α-granule (right panel). In addition, BDDFVIII was detected rarely within membrane systems in the platelet cytoplasm, indicating that the VWFSPD2 indeed had an increased efficiency to traffic BDDFVIII directly into the α-granule compartment.

**Figure 3:**
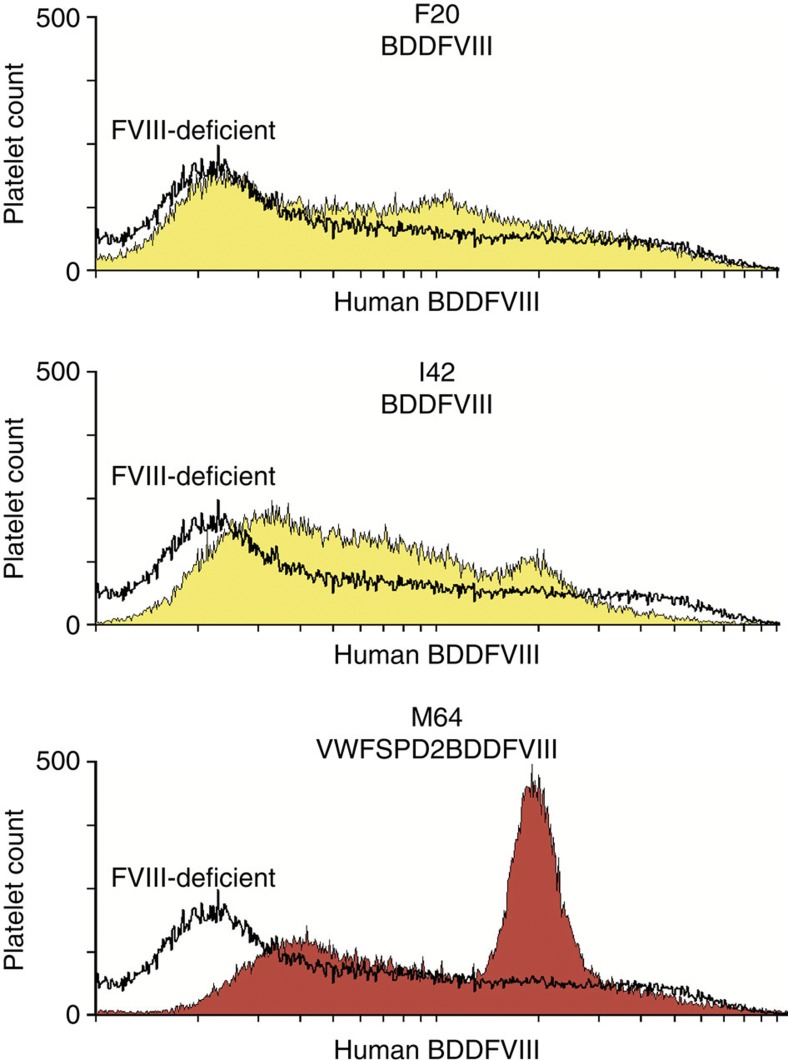
Quantitative analysis of platelet FVIII. Flow cytometric analysis of platelets. BDDFVIII was absent in platelets (*x* axis) analysed from a FVIII-deficient dog used as a negative control for staining with a human BDDFVIII 1°Ab and Alexa Fluor 568-conjugated 2°Ab (black, unshaded histogram). In contrast G-PBC from transplanted dogs displayed appreciable levels of platelet BDDFVIII (shaded histogram: −*889ITGA2B*-driven FVIII of F20 and I42, yellow; *−673ITGA2B-*driven FVIII of M64, red). The hierarchy for the mean fluorescence intensity of FVIII expression reveals that F20<I42<M64. Shown are the results from one experiment analysis of 50,000 platelets per sample at 2.9 (F20), 1.9 (I42) and 0.9 (M64) years after infusion of FVIII-transduced G-PBC, which is representative of the outcome of seven separate experiments.

Immunofluorescent flow cytometric analysis of platelets confirmed that M64 stored the greatest level of FVIII per platelet because M64 platelets displayed the highest mean fluorescent intensity for detection of FVIII followed by I42 and F20 compared with FVIII-deficient negative control platelets (Fig. 3). These results indicate that the VWFSPD2 targeting construct imparts an advantage for storing BDDFVIII within platelets. Subsequently, use of the smallest *−673ITGA2B* gene promoter allows the lentiviral vector to accommodate the largest therapeutic insert (in this case, the VWFSPD2–BDDFVIII), and therefore, may be more useful for gene transfer rather than the −1218 or −889 *ITGA2B* promoters.

**Figure 4:**
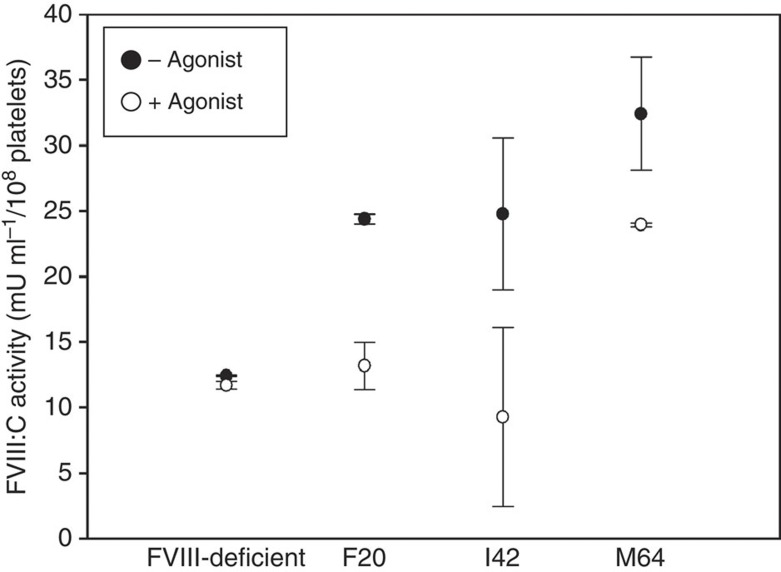
Activated platelets induced to secrete FVIII:C. To detect FVIII:C activity present in lysate of quiescent, untreated platelets (black circle; (−) agonist) and FVIII:C activity remaining in lysate after secretion of BDDFVIII from platelets treated with a mixture of physiological agonists of platelet activation: ADP, epinephrine and canine PAR1,3,4 (white circle: (+) agonist). Platelet lysates from a FVIII-deficient negative control dog show that the level of FVIII:C background activity is virtually unchanged for untreated and activated platelets. Dogs that received BDDFVIII-transduced G-PBC show an appreciable decrease in FVIII:C activity only after platelet activation; thus demonstrating that platelets isolated from experimental dogs can be induced to secrete FVIII. Each data point represents the mean value of FVIII:C activity from two independent samples each measured in duplicate with s.d. represented by black error bars. Shown is the result from one experiment performed at 2.7 (F20), 1.6 (I42) and 0.6 (M64) years after infusion of FVIII-transduced G-PBC, which is representative of the outcome of three separate experiments.

A Chromogenix Coatest SP_4_ FVIII assay was performed to determine whether activated platelets could secrete a biologically active form of BDDFVIII (FVIII:C) as previously shown for activated human megakaryocytes in tissue culture^12^. In Fig. 4, platelet lysates from a FVIII-deficient dog show that the level of BDDFVIII:C background activity is virtually unchanged for untreated (black, −agonist) and activated platelets (white, +agonist). In contrast, FVIII:C activity was detected readily in the lysate of quiescent, untreated platelets from F20, I42 and M64. Furthermore, BDDFVIII:C levels were decreased in lysates of platelets stimulated by a mixture of physiological agonists of platelet activation: ADP, epinephrine and canine PAR1,3,4 in all three experimental dogs. In summary, dogs that received BDDFVIII-transduced G-PBC show an appreciable decrease in FVIII:C activity only after platelet activation, suggesting that platelets from experimental animals can be induced to secrete FVIII within the vasculature.

### Genomic analysis of the lentiviral vector

**Figure 5:**
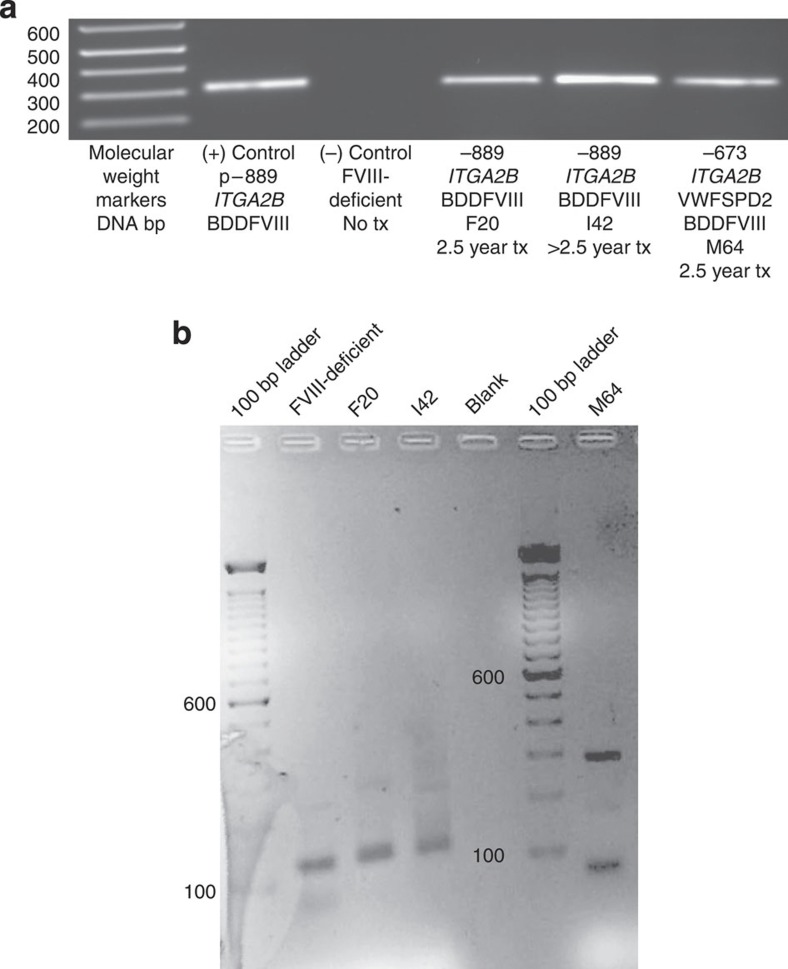
PCR analysis for detection and localization of lentiviral vector within canine genome. (**a**) Long-term detection of BDDFVIII-lentiviral vector within leukocyte genomic DNA. Shown is an ethidium bromide-stained agarose gel of PCR product-derived genomic DNA isolated from canine peripheral blood leukocytes. Molecular weight markers (Lane 1) are labelled on the left in base pairs (bp). In Lane 2, p−889*ITGA2B*-BDDFVIII-WPTS served as a positive control for PCR of a 302 bp region of the lentiviral WPRE. PCR failed to detect WPRE within leukocyte genomic DNA from a FVIII-deficient dog serving as a negative control (Lane 3). In contrast, WPRE was detected in the PCR of all three experimental dogs (F20, I42, M64; Lanes 4–6) for at least 2.5 years after G-PBC transplant demonstrating long-term insertion of the lentiviral vector into the canine genome. All reactions were run in triplicate from at least three separate samples collected periodically for at least 2.5 years after G-PBC transplant. (**b**) Linear amplification-mediated (LAM)-PCR to localize lentiviral vector within canine genome. Polyacrylamide gel of LAM-PCR products from a FVIII-deficient negative control and experimental dogs at 2.5 (F20), 1.4 (I42) and 0.5 (M64) years after infusion of FVIII-transduced G-PBC. PCR was performed on genomic DNA isolated from leukocytes purified with Ficoll from circulating peripheral blood. Each amplified band represents a distinct proviral integration. All lanes appear to contain multiple insertions, indicative of polyclonality. The panel underwent black–white inversion from the original digital image, and levels were globally adjusted to improve visibility of all bands. DNA standard markers (Lane 1 and 6) labelled on left in base pairs.

The lentiviral vector WPRE element was detected by PCR of genomic DNA isolated from leukocytes collected from F20, I42 and M64 for at least 2.5 years after transplant (Fig. 5a). Real time quantitative PCR (RT–qPCR) analysis of genomic DNA isolated from peripheral blood leukocytes revealed that the transduction efficiency for each lentiviral vector was 1% (F20), 4% (I42) and 2% (M64) (Table 1, column 8). The detection of lentiviral vector by genomic analysis in the absence of the appearance of insertional oncogenesis is consistent with the overall good health of all of the dogs with frequent evaluation of peripheral blood counts and peripheral blood smears documenting normal morphology and numbers of circulating hematopoietic cells. Linear amplification-mediated (LAM)-PCR was also performed to determine the integration pattern of lentiviral vector within the genome of the experimental dogs. Figure 5b shows that lentiviral vector was not present within the genome of a FVIII-deficient control, whereas multiple bands appear to be present in the genomic DNA of transplanted dogs (F20, I42 and M64). It is noteworthy that a distinct insertion site was detected specifically in chromosome 4 for F20 and chromosome 35 for M64. The results demonstrate that insertion of the lentiviral vector could be detected within I42 genomic DNA, although a site of insertion could not be localized to a precise region of the current canine genome map^34^. In summary, the results indicate that insertional mutagenesis had not occurred when this study was concluded (≈2.5 years after transplant). This is consistent with another report that found lentiviral vectors usually insert into benign areas of the genome in animals and humans^35^. However, oncogenesis as a result of mutagenesis due to random insertion of the lentiviral vector within the canine genome remains a potential risk with lentiviral gene transfer.

### Efficacy of platelet-targeted gene therapy for haemophilia A

**Figure 6:**
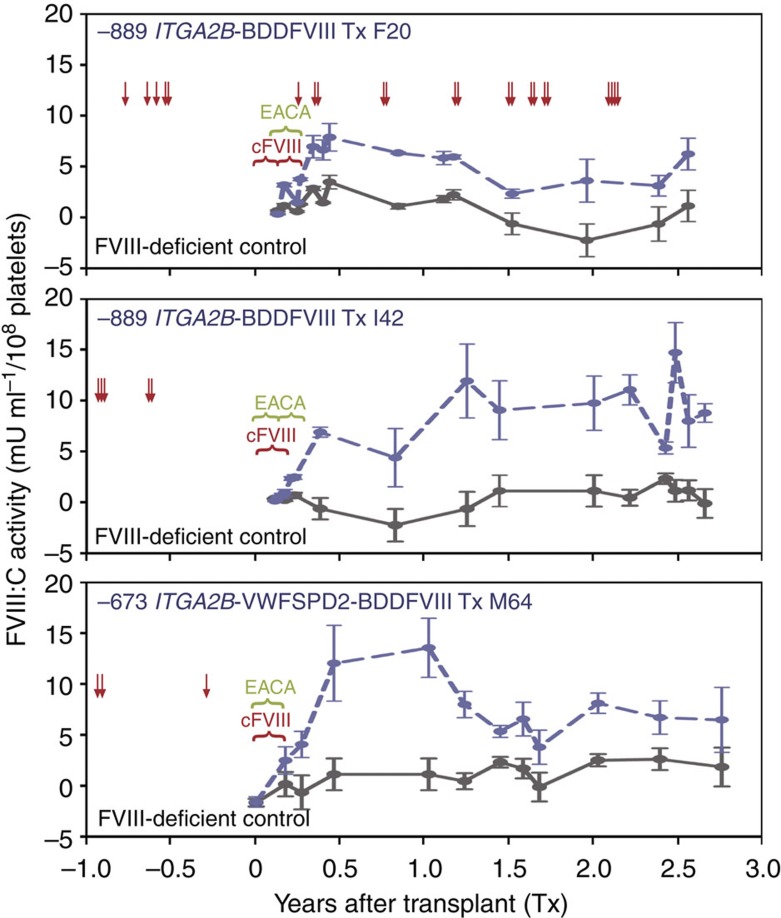
Correction of the canine haemophilia A phenotype with platelet BDDFVIII. A Coatest SP_4_ FVIII chromagenic analysis assay was performed on lysates of 1 × 10^8^ washed peripheral blood platelets resuspended in 1 ml of lysis buffer from samples collected periodically for at least 2.5 years after transplant (*x* axis). The mean value of FVIII activity (mU ml per 10^8^ platelets) is represented by the plotting of data points recorded from two independent samples (each measured in duplicate) with error bars showing ±s.d. for at least 12 time points (*y* axis). Analysis of platelet FVIII:C activity from a FVIII-deficient dog served as a negative control (black, solid line). Interestingly, the level of FVIII:C activity was detected above background levels at regular intervals (blue dashed line) for ≈2.5 years after transplant of BDDFVIII-transduced G-PBC (F20, top; I42, middle; M64, bottom panel). Note, each dog was administered daily injections of FVIII in the form of canine blood transfusions or cFVIII (red bracket) and EACA (green bracket) for uncontrolled bleeding for a short time after transplant (until haemocult tests were negative). As anticipated, each dogs required cFVIII supplements (red arrows) to help resolve severe bleeding episodes incurred before G-PBC transplant. Remarkably, only F20 (which had the lowest steady state level of FVIII:C activity) required cFVIII supplements after G-PBC transplant demonstrating platelet FVIII:C activity reached therapeutic levels in I42 and M64.

We observed previously that human hematopoietic cells could serve as a primary tissue source for the synthesis of a functional form of human BDDFVIII (FVIII:C) within tissue-cultured human megakaryocytes^12^,^36^, in peripheral blood platelets isolated from mice xenotransplanted with BDDFVIII-transduced human G-PBC^12^, and in a murine model for haemophilia A that received a transplant of BDDFVIII-transduced bone marrow^16^. The current study (Fig. 6) shows that FVIII:C activity (≈5–15 mU ml^−1^ per 10^8^ platelets) can be detected by chromogenic analysis for at least 2.5 years after autologous G-PBC transplant in each dog with the highest levels appearing ~1 year after transplant and typically leveling off to ≈5–10 mU ml^−1^ per 10^8^ platelets (F20, I42, M64; blue dashed line). Samples from FVIII-Deficient dogs served as negative controls for each time point (black line).

To determine the total level FVIII:C activity present within each animal at any given time we note that there is ≈2 × 10^8^ platelets per ml of blood and there is ≈92 ml blood per kg in dogs. Using values recorded in Table 1 for weight and transduction efficiency and the mean FVIII:C level of each dog calculated from data points shown in Fig. 6, it is estimated that there is ~0.230U (F20), 1.325U (I42) and 0.676U (M64) FVIII:C per dog stored within all of the circulating platelets. To put these values in perspective, the term 1U FVIII:C ml^−1^ defines 100% FVIII activity in the reference plasma from a normal (20 kg) dog; therefore, a normal (20 kg) animal has ≈800 total units of FVIII in its plasma volume at any given time.

The results in Fig. 6 show that multiple severe bleeding episodes occurred in each animal 1 year prior to G-PBC that required a transfusion with cFVIII supplements (red arrows). Note, to prevent bleeding due to the gene therapy protocol, each dog received daily supplements of cFVIII (red bracket) beginning on day 1 of the G-PBC transplant protocol. EACA (green bracket) was also administered to the transplanted dogs until blood was absent from their stool, which remarkably coincided with platelet FVIII:C levels reaching ≈5 mU ml^−1^ per 10^8^ platelets. Interestingly, F20 (top panel) displayed the lowest overall platelet FVIII:C levels of ≤5 mU ml^−1^ per 10^8^ platelets and also experienced severe intermittent bleeding episodes throughout the experimental follow-up of 2.5 years after transplant that required administration of additional supplements in the form of transfusions of normal canine plasma or cFVIII (red arrows). This result indicates that 5 mU ml^−1^ per 10^8^ platelets of FVIII:C appears to be a threshold level of transgene expression that must be overcome in canine haemophilia A to achieve adequate correction of the bleeding phenotype. Transplant dog I42 (middle panel) maintained the highest steady state of FVIII:C of ~9 mU ml^−1^ per platelets and did not experience severe bleeding requiring administration of cFVIII supplements, ultimately demonstrating correction of the haemophilia A phenotype for at least 2.5 years after transplant. Remarkably, M64 (bottom panel) reached 5 mU FVIII:C per ml per 10^8^ platelets earlier than the other transplant dogs with the synthesis of a hybrid SPD2FVIII molecule that obtained a mean FVIII:C activity level of 8 mU ml^−1^ per 10^8^ platelets. This result demonstrates that the use of either the −889ITGA2B gene promoter or the −673ITGA2B gene promoter coupled with the VWFSPD2 trafficking peptide can be used effectively to target BDDFVIII to platelets, leading to correction of the canine haemophilia A phenotype.

The time required for whole blood to clot in a test tube was measured for each dog using a traditional version of the Lee–White whole blood clotting time (WBCT) assay^37^. Haemostatically normal dogs have a mean WBCT of 10.5 min±s.d. 1.4 min. The baseline WBCT for the FVIII-deficient dogs was 44.5 (F20), 40.5 (I42) and >60 (M64) minutes before G-PBC transplant. After G-PBC transplant the average WBCT decreased to 39.5 (F20, *n*=5), 38.4 (I42, *n*=5) and 41.9 (M64, *n*=4) minutes. This result shows a very modest decrease in WBCT, which could be considered well within the normal variation of WBCT for FVIII-deficient dogs. Interestingly, this result supports our inability to detect FVIII:C within the plasma of the experimental dogs (which is an essential component for success of the WBCT). Thus, this outcome suggests that measurement of WBCT *ex vivo* is not a suitable assay to predict the efficacy for platelet FVIII to improve haemostasis *in vivo* because (unlike plasma FVIII) our results indicate that platelet-derived FVIII must be secreted from activated platelets following stimulation with physiological platelet agonists at the site of vascular injury to improve haemostasis within FVIII-deficient dogs as shown in Figs 4 and 6.

To determine whether the G-PBC transplant recipients developed a humoral antibody response to the newly expressed human BDDFVIII, canine blood plasma (from F20, I42 and M64) was screened for inhibitors with an activated partial thromboplastin time (aPTT) mixing assay that detects inhibitory antibodies to either coagulation factor VIII or IX. Plasma from haemophilia A dogs with known Bethesda inhibitor (BIU) titres that crossreact with and inhibit human FVIII was used as a positive control, and plasma from dogs without inhibitors was assayed concurrently as a negative control for comparative analysis. Our results indicate that F20, I42 and M64 did not develop inhibitors (Table 1: column 12). This result is consistent with our inability to detect the presence of FVIII:C in the plasma. This outcome is identical with the failure of haemophilia A mice to develop inhibitory antibodies to the human platelet BDDFVIII and our the inability to detect FVIII:C in the plasma following transplant of lentiviral vector-transduced murine bone marrow^16^. This further supports our hypothesis that targeting transgene synthesis of BDDFVIII to platelets may be a potentially effective treatment for humans with pre-existing antibodies to FVIII (refs 15, 38).

## Discussion

Recent reports indicate that haemophilia A may be ameliorated by targeting expression of human FVIII to the liver by intravenous infusion of a new generation of adeno-associated viral (AAV) vectors that appears to show feasibility for restoring haemostasis in haemophilia A patients^32^,^39^. However, AAV clinical trials targeting liver will likely exclude haemophilia patients with pre-existing liver damage due to the acquisition of hepatitis or HIV from contaminated blood products from factor replacement therapy, or those individuals who have developed inhibitory antibodies to plasma FVIII or pre-existing immune responses to the AAV capsid. Thus, an *ex vivo* approach using the hematopoietic stem cell as a target for lentiviral gene transfer of FVIII may be an ideal strategy for ~30% of patients with underlying conditions that exclude them from liver-targeted gene transfer protocols. Although, it remains to be determined whether a generalized expression of FVIII within all hematopoietic cell lineages is sufficient for ecotopic expression of FVIII to correct haemophilia A in humans^40^, or if the strategy presented within this report targeting expression specifically to platelet α-granules has an added advantage of delivering FVIII directly at the site of the vascular injury. As the platelet-targeted approach prevents protein expression within other cell lineages, it may prevent leakage of FVIII into the blood plasma by cells that do not store FVIII within granules or expression of FVIII within antigenic cell-types that could potentially elicit a response from the immune system.

From a quantitative perspective, use of platelet-targeted synthesis of FVIII results in the production of 0.2-1.3 U FVIII:C per dog. Although this concentration of FVIII is very modest compared with the level of FVIII within the plasma of normal dogs, the data show that our approach is sufficient to provide therapeutic benefit for I42 and M64 for at least 2.5 years after G-PBC transplant (please see records of post-transplant bleeding events depicted in Table 1 (column 11) and Fig. 6). This is consistent with the observation that from a cofactor perspective, in the presence of calcium and phospholipids even trace amounts of FVIII can increase the conversion of FX to FXa by FIXa to levels with therapeutic effects. Thus the outcome of this study indicates that secretion of FVIII by platelets precisely at the site of the vascular injury is likely be a feasible and clinically relevant strategy for establishing haemostasis in patients affected with haemophilia A.

It is important to note that the lack of spontaneous formation of antibodies to human FVIII is not necessarily evidence of immune tolerance induction. The absence of inhibitory antibodies may be the direct consequence of ‘sequestering’ the FVIII (which is normally a plasma protein) within platelet α-granules. Furthermore, the sub-myeloablative condition regimen utilized to create a niche in the bone marrow for the engraftment of the lentiviral vector-transduced G-PBC may have played a role towards our inability to detect inhibitory Ab to human BDDFVIII. This is supported by our previous report that transient immune suppression with intravenous immunoglobulin, cyclosporine and prednisone were used to successfully diminish an immune response that developed in a GT dog transplanted with G-PBC transduced with a lentiviral vector expressing ITGA2B on platelets^19^. This is consistent with another report where immune suppression helped diminish an immune response in non-human primates that developed inhibitory antibodies to human FVIII (ref. 39). Clearly, the strategy for platelet FVIII gene transfer does not appear to elicit the formation of inhibitory antibodies, although this topic requires further study.

Interestingly, (following G-PBC pre-transplant and conditioning) F20 successfully fathered a litter of pups affected with haemophilia A with no other detectible abnormalities. As use of chemotherapeutic agents for pre-transplant condition and transient immune suppression carries the risk of side-effects and sterility, data from this study suggest that the particular pre-transplant conditioning regimen with busulfan followed by a short course of immune suppression with MMF and CSP used in this study does not appear to affect the ability of the dog to produce viable offspring. Note, I42 and M64 received a lower dose of busulfan preconditioning than F20 (Table 1, column 2), yet it remains to be determined whether these dogs have also remained fertile because they were not used to father litters after gene transfer. Although, the likelihood that the single dose pre-transplant conditioning regimen did not affect fertility is consistent with well-established clinical information from patients that received a single dose of conditioning with busulfan^41^,^42^.

Collectively, our results demonstrate that hematopoietic G-PBC gene transfer provides long-term correction of the haemophilia A bleeding phenotype in dogs. This outcome is consistent with our previous studies showing successful expression of FVIII within a human transformed megakaryocyte cell line, and the feasibility of platelet factor VIII to restore haemostasis in the murine haemophilia A^15^,^16^. The current work demonstrates feasibility of targeting BDDFVIII specifically in platelets to improve haemostasis within a canine ‘large-animal’ model of haemophilia A. This work indicates precedence for proposing trials to determine whether this clinically relevant strategy demonstrated herein for targeting BDDFVIII into platelets has the ability to provide therapeutic benefit for human haemophilia A patients. This study also paves the way for better management of patients with other inherited platelet bleeding diseases and potentially a wide spectrum of disorders because platelets have a role in many cellular processes^1^.

## Methods

### Human transformed cell lines

Pro-megakaryocytic (HEL)^43^,^44^, T-cell lymphoma (KT1)^45^, B-cell lymphoma (Raji)^46^, erythroleukemia (K562)^47^, and epithelial (HeLa)^48^ cells were obtained from American Type Culture Collection (Rockville, MD, USA).

### Luciferase reporter gene promoter vectors.

*ITGA2B* Gene Promoter Constructs: Genomic DNA was isolated from the human pro-megakaryocyte cell line, Dami^49^, and human *ITGA2B* gene promoter fragments were amplified by PCR using either sense primer ‘−1218’(5′-TTACGCGTCGACAGATCT**AAATGTGGCTGGTTACCCC**-3′)‘−1198’ (bold) of *ITGA2B* or ‘-889’(5′-TTACGCGTCGACAGATC**TGTGCTCAATGCTGTGCC**-3′)‘-872’ (bold) of *ITGA2B* or ‘−673’(5′-TTACGCGTCGACAG**ATCTCCTTGCCACCTAGACC**-3′)‘−654’ (bold) of *ITGA2B* and anti-sense primer (5′-GGCGTCTTCCATGG**TCCTTCTTCCACAACC**-3′) encoding nucleotides +99 to +86 of luciferase pGL3-BASIC and nucleotides +30 to +15 (bold) of *ITGA2B* gene promoter. Correct identity of constructs was confirmed by nucleotide sequence analysis.

pCMVLuc: A *Bgl*II and *Hin*dIII restriction digest of cytomegalovirus tissue nonspecific gene promoter (878 bp) from pRc/CMV (Invitrogen) is ligated into the pGL3-Basic Luciferase vector (Promega, Madison, WI). This construct served as the positive control for high level gene expression within all cell types, thus, assigned an arbitrary level of 100% luciferase activity for each cell line (Fig. 1).

pGL3-BasicLuc: negative control construct for 0% luciferase activity (Fig. 1) because lacks a gene promoter to drive luciferase gene transcription (Promega).

pCMVnlac: Cell lines were co-transfected with one of the pITGA2BLuc+ constructs and pCMVnlac encoding the β-galactosidase marker gene to normalize transgene expression^49^.

### Luciferase gene promoter reporter assay

Cell lines (2 × 10^7^) were co-transfected with either (20 μg) of the *ITGA2B* gene promoter construct (−1218, −889, −673) (Fig. 1a) or the positive (CMV) or negative (Basic) controls encoding firefly luciferase and pCMVnlac (20 μg) encoding β-galactosidase^49^. Briefly, 48 hours after co-transfection cells were washed, harvested and lysates were prepared and frozen to −80 °C using the luciferase assay system (Promega). Luciferase activity was measured with a Turner Designs Model 20 Luminometer. Detection of β-galactosidase activity was performed to normalize transient transgene expression for each cell line with a sensitive ELISA enzymatic assay that measured colormetric change with the substrate for β-galactosidase, chlorophenol red β-D-galactopyranoside (CPRG)^50^. The percent of luciferase activity was determined by comparing the mean value of the relative light units (RLU) of luciferase/CPRG Vmax value for each construct to reveal the transfection efficiency for each cell line. The RLU for pCMVLuc was assigned arbitrarily a value of 100% and all other results were calculated for each vector based upon that value as shown in Fig. 1a.

### *ITGA2B* promoter-driven lentiviral vector for human BDDFVIII

*ITGA2B*- WPTS genetic transfer vectors are derived from a HIV type-1 lentiviral vector (D.Trono, University of Geneva, Switzerland)^51^. p−889ITGA2B-BDDFVIII-WPTS lentiviral vector (Fig. 1b) encodes a −889 to +30 nucleotide fragment of the human *ITGA2B* promoter and human BDDFVIII molecule^16^. p−673ITGA2B−VWFSPD2-BDDFVIII-WPTS lentiviral vector (Fig. 1c) encodes a fragment of the human *ITGA2B* gene promoter from nucleotide −673 to +30 followed by a fragment of the human Von Willebrand Factor propeptide (VWFpp) encoding the VWF signal peptide (SP;66 bp) linked to the D2 domain(1,199 bp) and cDNA encoding human BDDFVIII to allow megakaryocyte-specific transcription of a hybrid molecule that uses the SPD2 peptide to traffic human BDDFVIII to platelet α-granules^22^. cDNA encoding SP was amplified by PCR with forward primer (P)1 (5′-GTTAATCGATATCTCCTTGCCACCTAGA-3′) and reverse P2 (5′-AATCTGGCAGGAATCATGGTCCTTCTTCCACAACCT-3′) and ligated to D2 amplified by PCR using forward P3 (5′-AGGTTGTGGAAGAAGGACCATGATTCCTGCCAGATTTGC-3′) and reverse P4 (5′-CGTCTCGGCCCTTTTGCTGCCATGAGACAG-3′). A nested PCR-linked *ITGA2B* promoter and VWFSPD2 with P5 (5′-ATCGATATCTCCTTGCCACCT A-3′) and P4. p−889ITGA2-BDDFVIII-WPTS served as a template for PCR of cDNA encoding a fragment of BDDFVIII using forward P7 (5′-CGTCTCAGGGCCACCAGAAGATACTACCT-3′) and reverse P8 (5′-ACGCGTCTTCTCTACATACTAGTA-3′) to synthesize cDNA that ligated directly to VWFD2. All PCR products were cloned into pCR-Blunt II-TOPO (Life Technologies, Grand Island, NY) using unique restriction sites −673ITGA2B-SPD2(*Cla*I and *Bsm*BI) ligated to 5′hBDDFVIII (*Bsm*B1 and *Mlu*I) with 3′BDDFVIII (*Mlu*I and *Spe*l). All fragments were cloned into pWPTS lentiviral vector and the correct identity confirmed by nucleotide sequence analysis. Recombinant virions were generated from three-plasmid transient co-transfection followed by supernatant collection, 500-fold concentration by centrifugation and storage at −80 °C until utilized^19^. Virion titre was determined by RT–PCR^52^. Replication-competent virions were confirmed absent from stocks with marker rescue assays^26^.

### Dogs

Cytokine mobilized CD34+G-PBC gene transfer and autologous transplant studies using FVIII-deficient dogs affected with haemophilia A (University of North Carolina, Chapel Hill, NC)^4^ were conducted and approved by Institutional Animal Care and Use Committees of the University of North Carolina and The Medical College of Wisconsin, which are both accredited facilities of the American Association for Accreditation of Laboratory Animal Care.

### CD34+ G-PBC isolation and transplantation of transduced cells

Adult (1.25, 4.25 and 6.5-year-old) FVIII-deficient male dogs were injected daily with canine recombinant granulocyte colony stimulating factor (crG-CSF; 10 μg kg^−1^ d^−1^) and stem cell factor (crSCF; 5 μg kg^−1^ d^−1^) (Amgen, Thousand Oaks, CA, USA). G-PBC collection was performed on the third day using a COBE Spectra Blood Cell Separator. Mononuclear G-PBC were isolated with Fico-Paque Plus (GE Healthcare, Uppsala, Sweden). CD34+ G-PBC were selected with a biotin-conjugated-1H6 Ab (1 mg ml^−1^) (Richard Nash, Fred Hutchinson Research Institute, Seattle, WA, USA) and anti-biotin immuno-magnetic beads (1:5 dilution) on an Automacs magnetic cell separator (Miltenyi Biotec Inc., Auburn, CA, USA).

CD34+G-PBC were transduced with −889ITGA2B-BDDFVIII-WPTS or −673ITGA2B-VWFSPD2-BDDFVIII-WPTS lentiviral vector. Briefly, 4 × 10^6^ cells per well were seeded in a six-well plate (Falcon-Becton Dickinson, Franklin Lakes, NJ, USA) coated with 20 μg cm^−2^ RetroNectin (Takara Shuzo, Otsu, Shiga, Japan) and incubated with 1.0 × 10^4^ *ITGA2B*–FVIII lentivirions per cell in X-Vivo 10 containing 10% FCS, rhIL-3, rcaIL-6, rcaSCF, rhTPO and rhflk2/flt3 ligand. Approximately 3 × 10^6^ FVIII-transduced CD34+G-PBC per kg and 2 × 10^8^ CD34(−) G-PBC were infused into the cephalic vein of each autologous transplant recipient pre-conditioned with a nonmyeloablative dose of 5-10 mg per kg Busulfex. Transient immune suppression administered for ≈90 days after transplant with 10 mg kg^−1^ per day cyclosporine (Gengraf, Abbott Laboratories, North Chicago, IL, USA) and 8 mg kg^−1^ per day MMF (Table 1)^19^.

### Blood collection

Blood was collected at preselected times into a vacutube containing 7.5% EDTA anticoagulant^19^. Blood cells were counted on a Vet ABC haematology analyser (scil animal care company, Gurnee, IL, USA). Platelets were isolated with Fico/Lite (Atlanta Biologicals, Norcross, GA, USA), washed with PBS and used directly for immunofluorescent flow cytometry or FVIII:C activity analysis. Leukocytes were isolated with Ficoll-Paque Plus (GE Healthcare) according to the manufacturer’s specifications.

### Antibodies

A murine monoclonal 1°Ab to canine CD34 ‘1H6’ (1 mg ml^−1^) was from the Fred Hutchinson Cancer Research Center (Seattle, WA, USA)^27^. A sheep anti-rabbit fibrinogen polyclonal 1°Ab (5 μg ml^−1^) that recognizes canine fibrinogen was purchased from Enzyme Research. Monoclonal 1°Abs (5–10 μg ml^−1^), MBC 103.3 and 301.3 (R.R. Montgomery, BloodCenter of WI, Milwaukee, WI, USA) recognize epitopes on human BDDFVIII (ref. 53). 2°Abs used were Alexa Fluor 488 F(ab′)_2_ conjugated to a fragment of donkey anti-sheep IgG (H+L) (1:1,000 dilution) and Alexa Fluor 568 F(ab′)_2_ fragment of goat anti-mouse IgG (H+L) (1:500 dilution) were from Life Technologies (Grand Island, NY, USA).

### Immunofluorescent confocal microscopy

Canine platelets were fixed with 3.7% (vol/vol) buffered formalin, permeabilized in 0.5% Triton X-100 (in 20 mmol l^−1^ Hepes, 300 mmol l^−1^ sucrose, 50 mmol l^−1^ NaCl and 3 mmol l^−1^ MgCl2, pH 7.0) and blocked with 2.5% normal goat serum in HBSS. Platelets were incubated with a sheep polyclonal 1°Ab to canine fibrinogen and monoclonal 1°Ab (MBC 103.3 and 301.3) to human FVIII (5 μg ml^−1^) overnight at 4 °C^53^. The Alexa Fluor 488-conjugated F(ab′)_2_ fragment of donkey anti-sheep IgG (H+L) was used as a 2°Ab (1:1,000 dilution) to detect fibrinogen and Alexa Fluor 568-conjugated F(ab′)_2_ fragment of goat anti-mouse IgG (H+L)-conjugated 2°Ab (1:500 dilution) was used to detect the presence of FVIII for 30 min at 25 °C. Platelets were mounted with Vectashield (Vector Labs, Burlingame, CA, USA). Immunofluorescence was detected with a Zeiss LSM 510 Multiphoton Confocal Microscope (Carl Zeiss, Inc. Oberkochen, Germany)^54^. Platelets isolated from FVIII-deficient dogs were used as negative controls. Nonspecific isotype control Ab served as negative controls. Platelets were imaged by Z sections taken for each field and the entire Z series (12-25 images) combined into a stacked projection. The projections were merged using the Confocal Assistant software programme (Bio-Rad). Computer-assigned colours were based on the intensities of bitmap overlaps, with Alexa488-fluorochrome represented by green pixels, Alexa568-fluorochrome represented by red pixels and colocalization of the two fluorochrome-conjugated antibodies represented by yellow pixels.

### Immunofluorescent flow cytometry

Canine platelets were isolated from blood and treated with Cytofix and PERM/WASH reagents (BD Biosciences) for intracellular detection of BDDFVIII. Platelets were incubated with a monoclonal 1°Ab (MBC 103.3 and 301.3) to human FVIII (5 μg ml^−1^) 30 min at 4 °C and then incubated with Alexa Fluor 568-conjugated F(ab′)_2_ fragment of goat anti-mouse IgG (H+L) conjugated 2°Ab (1:500 dilution) for 30 min at 4 °C. Platelets isolated from FVIII-deficient dogs were used as negative controls. Nonspecific isotype control Ab served as negative controls. Cells were collected and analysed on an Accuri C6 Flow Cytometer (Accuri Cytometers, Inc., Ann Arbor, MI, USA) using the Accuri analysis software.

### Immunogold labelling

Platelets were fixed in 1.25% glutaraldehyde (Fluka AG, Buchs, Switzerland), infused with 2.3 M sucrose (Fluka) and frozen with a Reichert KF 80 freezing system (Leica, Vienna, Austria). Sections of ≈80 nm were prepared with the Ultracut E ultramicrotome equipped with a FC 4E cryokit attachment and placed on collodion-coated nickel grids. Grids were incubated for 10 min on PBS with 1% BSA and then placed on (10 μg ml^−1^) drops of the 1°Ab to FVIII (301.3) for 1 h at 25 °C. Sections were incubated for 1 h with a goat anti-mouse 2°Ab adsorbed onto 10-nm gold particles (1/100 dilution of AuroProbe EM G10). Controls included the use of an irrelevant IgG of the same species and at the same concentration.

### Electron microscopy

Grids were stained by uranyl acetate and osmium and then embedded in methylcellulose prior to observation with a Jeol JEM-1010 transmission electron microscope (Jeol, Croissy-sur-seine, France) at 80 KV.

### Agonist-induced activation of platelets

Platelets were isolated from circulating peripheral blood, washed and activated with physiological agonists of platelet activation. To induce activation, platelets were resuspended in Tyrode’s buffer (2.5 × 10^6^ per ml) containing 1 mM CaCl_2_, 1 mM MgCl_2_, 25 μM each of adenosine diphosphate (ADP) (Sigma), epinephrine (Bio/Data Corporation, Horsham, PA, USA) and canine thrombin receptor activating peptides (synthesized in our core laboratory): PAR1 (SFFLKN-NH2), PAR3 (TRFGAP-NH2) and PAR4 (SFPGQP-NH2) for 30 min at 37 °C as previously described^19^,^31^. Separate aliquots were incubated in Tyrode’s buffer without agonist as a negative control. The platelets were pelleted by centrifugation and supernatant was aspirated and discarded from agonist-treated and negative control samples. The platelet pellet was frozen immediately to −80 °C until being tested for FVIII:C activity using the coatest assay.

### PCR detection of lentiviral vector in blood genomic DNA

DNA was isolated with a QIAamp DNA Blood Mini Kit (Qiagen, MD, USA) from canine leukocytes purified with Ficoll-Paque Plus (Amersham Pharmacia Biotech AB, Uppsala, Sweden). p−889ITGA2-BDDFVIII-WPTS served as a positive control. PCR analysis was performed with Taq polymerase (Invitrogen, Carlsbad, CA, USA) on a PTC200 instrument (MJ Research, Watertown, MA) with forward primer P1 (5′-ACGCTATGTGGATACGCTG-3′) and reverse primer P2 (5′-AACACCACGGAATTGTCAG-3′) to synthesize a 318 nucleotide primary product encoding the WPRE (Fig. 1b,c). A secondary PCR reaction was performed with nested forward primer P3 (5′-TGGATACGCTGCTTTAATGC-3′) and reverse primer P4 (5′-AATTGTCAGTGCCCAACAG-3′) encoding a 302 bp product of WPRE (Fig. 5a).

### RT–qPCR to detect lentiviral transduction efficiency

Percent lentiviral gene marking was measured by RT–qPCR using Bio-Rad CFX96 Real-Time System^52^. Briefly, 12.5 μl of TaqMan Universal PCR Master Mix (Life technologies), a 900 nM concentration of each primer and 200 nM probe were combined in 20 μl of water. Then, 5 μl of canine genomic DNA was added and PCR was performed as follows: 2 min at 50 °C, 10 min at 95 °C, and then 40 cycles of 15 s at 95 °C and 1 min at 60 °C. For each RT–qPCR, a no-template control was included as negative control. Each sample was analysed in triplicate for gene copy number using Primer Express software (version 1.0; Applied Biosystems) and the mean value for transgene copy number/genome was converted to percent peripheral blood cells positive for lentiviral vector (also known as transduction efficiency) and reported in Table 1 (column 8). The lentiviral LTR primers and probe used were: forward: 5′-AGCTTGCCTTGAGTGCTTCA-3′; reverse: 5′-TGACTAAAAGGGTCTGAGGGA-3′; probe: 6FAM 5′-TGCCCGTCTGTTGTGTGACTCTG-3′ MGBNFQ. The canine *ITGB3* gene was used as an endogenous control for gene copy number with forward :5′-ATGCATCCCACTTGCTGGTAT-3′; reverse: 5′-TGCCCATCGTTAGGTTGG-3′; probe: 6FAM5′-TGCCTGCCAGCCTTCCATCCAG-3′ MGBNFG. Copy number was based on TaqMan principle. Ten-fold serial dilution of the plasmid constructs of known concentration containing relevant sequences (lentiviral vector LTR and canine *ITGB3*) were used to create standard curves for quantification of samples.

### Linear amplification-mediated PCR

LAM–PCR was performed to localize the lentiviral vector insertion sites within genomic DNA isolated from peripheral blood leukocytes. Briefly, the junction between integrated proviral LTR and the host genome was selected by two rounds of linear PCR (95 °C for 5 min (95 °C for 1 min, 60 °C for 45 s, 72 °C for 90 s) × 50; 72 °C for 10 min) with a vector-specific 5′-biotinylated primer (5′-/biotin/-GAACCCACTGCTTAAGCCTCA-3′) and purified using streptavidin-coated magnetic beads (Dynal M-280). Products were double-stranded using Klenow polymerase and random hexanucleotide primers and digested with *Tsp*509I at 65 °C for 2 h. Directional double-stranded linker oligos were ligated onto the non-LTR end and the resulting products were amplified by nested PCR (95 °C for 5 min (95 °C for 1 min, 60 °C for 45 s, 72 °C for 90 s) × 35; 72 °C for 10 min) using LTR-specific forward primers (F1: 5′-/biotin/-AGCTTGCCTTGAGTGCTTCA-3′; F2: 5′-AGTAGTGTGTGCCCGTCTGT-3′) and linker cassette specific reverse primers (R1: 5′-GACCCGGGAGATCTGAATTC-3′; R2: 5′-AGTGGCACAGCAGTTAGG-3′). Between rounds of nested PCR, products were purified using streptavidin-coated magnetic beads. Products were visualized on 2% TAE agarose gels. For sequencing, products were gel-purified and cloned into pCR2.1-TOPO, transformed into *E. coli* Top10, selected on LB-Amp-Xgal plates and amplified by colony PCR using M13F/R.

### Functional assessment of integration sites

Sequence products from LAM–PCR that were verified to contain proviral LTR sequence were masked for known genomic repeats and proviral features. The resulting sequence was aligned to the dog genome (CanFam 2.0, May 2005 assembly) using the Blat (BLAST-like alignment tool) server at UCSC. Sequences mapping to a unique location in the genome at 95% similarity were selected and integration sites were determined as the base in the genomic alignment flanking the proviral LTR sequence. For each site, the closest RefSeq gene was determined and compared with a list of human cancer orthologs.

### Detection of biologically active human FVIII (FVIII:C)

Lysates of 1 × 10^8^ platelets per ml were tested for FVIII:C using a Chromogenix Coatest SP_4_ FVIII kit (DiaPharma, Franklin, OH, USA)^12^. Duplicate samples of supernatant were placed in uncoated wells of a 96-well microtiter plate (25 μl per well) and assay components (phospholipid, Factor IXa, Factor X and calcium chloride) were added and incubated for 10 min at 37 °C. The chromogenic Factor Xa substrate S-675 was added and the plate was transferred to a Wallac Victor^2^ microplate reader preset at 37 °C. The Factor Xa-dependent conversion of S-2675 is directly related of the amount of FVIII:C in each well. A standard curve was constructed by plotting known amounts of recombinant human FVIII (Kogenate; Bayer Healthcare Pharmaceuticals, Berkeley, CA, USA) diluted in platelet lysate buffer using *V*_max_ at 405 nm. The *V*_max_ of each reaction was converted to units of FVIII:C activity using the kinetic software programme, SOFTmax, v.2.34 (Molecular Devices). The FVIII activity was measured by an endpoint reading at 405 nm, a background reading at 490 nm was subtracted from 405 nm. The total maximum FVIII:C per dog was calculated by multiplying the mean FVIII:C U ml per 1 × 10^8^ platelets × 92 ml blood/kg × dog weight (kg) × 2 × 10^8^ platelets per ml of blood using measured values recorded in Table 1 and Fig. 6.

### WBCT assay

WBCT is a modification of the Lee–White clotting time using two siliconized glass tubes (Becton Dickinson, Rutherford, NJ) at 28 °C (ref. 37). One ml of whole blood was drawn and 0.5 ml blood was distributed into each tube. A timer was started. After one minute, one tube was tilted every 30 s, the other left undisturbed. When a clot formed in the tilted tube, the second tube was then tilted every 30 s until a clot formed. The time for formation of a fully gelled clot in the second tube was recorded as the WBCT. Blood was collected from a haemostatically normal (WBCT 7.5-12.5 min) and the three experimental dogs (F20, I42 and M64) before and after G-PBC transplant if animals had not been treated with plasma for at least 1 month.

### Inhibitor assay to detect immune response to human FVIII

Canine blood plasma (F20, I42 and M64) was screened for inhibitors with an aPTT mixing assay that detects inhibitory antibodies to either coagulation factor VIII or IX as previously described^55^,^56^,^57^. Briefly, test plasmas are incubated in a 1:1 mix with normal plasma for 2 h at 37 °C and then the incubated mixture is analysed using standard aPTT reagents. Plasma from haemophilia A dogs with known BIU titres that crossreact with and inhibit human FVIII (positive control) and plasma from dogs without inhibitors (negative control) were assayed concurrently for comparison.

## Author Contributions

D.A.B., E.S.J., E.M., R.A.R., J.F., S.B.K., P.M.J. and Q.S. performed experiments and analysed data. L.M.D., P.N., A.T.N., T.C.N., S.L.H., T.B.H., K.C. and D.A.W. performed experiments, analysed data and helped write the manuscript.

## Additional information

**How to cite this article:** Du, L.M. *et al.* Platelet-targeted gene therapy with human factor VIII establishes haemostasis in dogs with haemophilia A. *Nat. Commun.* 4:2773 doi: 10.1038/ncomms3773 (2013).

## Figures and Tables

**Table 1:** Conditions for Autologous Transplant of *ITGA2B*-BDDFVIII Transduced CD34+ G-PBC into FVIII-Deficient Dogs.